# Stretchable and Washable Electroluminescent Display Screen-Printed on Textile

**DOI:** 10.3390/nano9091276

**Published:** 2019-09-07

**Authors:** Daniel Janczak, Marcin Zych, Tomasz Raczyński, Łucja Dybowska-Sarapuk, Andrzej Pepłowski, Jakub Krzemiński, Aleksandra Sosna-Głębska, Katarzyna Znajdek, Maciej Sibiński, Małgorzata Jakubowska

**Affiliations:** 1 The Institute of Metrology and Biomedical Engineering, Faculty of Mechatronics, Warsaw University of Technology, A. Boboli 8 St., 02-525 Warsaw, Poland (M.Z.) (T.R.) (L.D.-S.) (A.P.) (J.K.) (A.S.-G.); 2 Department of Semiconductor and Optoelectronics Devices, Faculty of Electrical, Electronic, Computer and Control Engineering, Lodz University of Technology, Żeromskiego 116 St., 90-924 Lodz, Poland (K.Z.) (M.S.) (M.J.)

**Keywords:** wearable electronics, stretchable polymer composite, printed electronics, screen-printing

## Abstract

Stretchable polymer composites are a new group of materials with a wide range of application possibilities in wearable electronics. The purpose of this study was to fabricate stretchable electroluminescent (EL) structures using developed polymer compositions, based on multiple different nanomaterials: luminophore nanopowders, dielectric, carbon nanotubes, and conductive platelets. The multi-layered EL structures have been printed directly on textiles using screen printing technology. During research, the appropriate rheological properties of the developed composite pastes, and their suitability for printed electronics, have been confirmed. The structure that has been created from the developed materials has been tested in terms of its mechanical strength and resistance to washing or ironing.

## 1. Introduction

The development of clothing integrated electronics requires technology that will not affect its operating conditions [1,2,3]. Integrated devices must be flexible and stretchable, in order to provide adequate comfort of use. In most studies, factors such as washing or ironing, that could damage the device, were not considered. In this paper, the example of a display, integrated with a T-shirt, is presented. Flexible electroluminescent structures have considerable economic potential, due to their properties and low production costs [4]. Examples of applications, include panel backlights, advertisements, and decorations [5].

In wearables, these screens are usually made, using various printing methods, such as inkjet or screen printing [6,7]. They are made by applying appropriate functional layers to the substrate. The display consists of two layers of conductive electrodes, with an insulating dielectric layer and a luminophore between them. The layers are usually printed with composites of dissolved elastic polymer and filler powders. The electrodes produced in this technique often require high voltage in the range of 100–1000 V and various frequencies [8,9,10]. Therefore, the insulating layers should be added to the application to ensure users’ safety.

In the study of Yakoh et al. [5], the display was made on Poly(ethylene terephthalate) (PET) film, and subsequent layers were made using screen printing. It has been shown that the display still works during bending. The use of foil, as a substrate, makes it difficult to integrate with clothing. PET film does not allow stretching or bending with a very small radius.

Another attempt was made, using Poly(3,4-ethylenedioxythiophene):poly(styrene sulfonic acid) coated polyester fabric using a three-dimensional (3D) printing method [11] and was found to be capable of uneven electroluminescence. Also, using polyester fabric, Verboven et al. [12] were able to achieve event and bright electroluminescence, using a screen printing method. A polyester fabric was used instead of cotton in both studies, which made it impractical for everyday applications and wearable electronics.

Different approaches were applied using a self-powered motion-driven triboelectric electroluminescence system, where an electroluminescent display was powered by friction between specially prepared and woven threads [13]. Such displays could be easily incorporated in clothing, although, it is not noticeable in bright light with their very low luminescent intensities.

Attempts to make the display on textiles were made by Słoma et al. [6], where layers of display were printed directly on the cotton fabric. The printing inks, used for the deposition, were based on a carrier of Poly(methyl methacrylate) (PMMA). While, bending the display resulted in uneven lighting and finally its physical damage.

Most of currently developed printed displays do not meet the conditions of clothing usage. In this work, the authors focused on producing a display that is flexible, does not degrade during washing and ironing and can be easily integrated with clothing. The results of this study will help to integrate new applications with clothing. Some of them were previously made using screen printing, such as radio-frequency identification antennas [14] or sensing materials [15], that could find its use in wearable electronics and in medical studies.

## 2. Materials and Methods

### 2.1. Materials

Thermoplastic polyurethane polymer (TPU) was selected to produce the organic base for the stretchable composites. This polymer was characterized by good mechanical properties, high optical transparency, and good solubility in various organic solvents. Commercially available TPU with density 1.18 g/cm^3^ from BASF company, was used as a solution in a mixture of Dimethylformamide (DMF) and Tetrahydrofuran (THF) in a 2:1 ratio. The dissolution process was conducted with a magnetic mixer for 2 h, at a controlled temperature of 40 °C. A solution containing 15 wt.% to 20 wt.% of polymer has rheological properties suitable for screen printing technology.

During the tests, several types of fillers were used for the preparation of electrical layers. Silver flakes and graphene nanoplatelets were used as the materials of the functional phase of the conducting layers. Graphene nanoplatelets, acquired commercially from CheapTubes, were characterized by average particle thickness of 10 nm and an average diameter of 15 μm. As the functional phase, which affects the high conductive character of printed layers, silver flakes AX 20LC, with average particle sizes 2–4 µm, from Amepox Company were used.

Dielectric layers were made of a composite containing barium titanate (BaTiO3) powder (particles size of 0.2 μm) from Inframat Advanced Materials (IAM, Manchester, CT, USA). Luminescence layers were prepared from blue luminophore powder from Osram-Sylvania, it consisted of ZnS:Cu grains with a mean powder diameter of around 45 nm.

The transparent electrode contained oligowalled carbon nanotubes CNT (from Cheap Tubes Inc.). Nanotube diameter was estimated in the range of 10–160 nm, and their length was between 0.5 and 5 μm. Additionally, as a transparent electrode, the authors used commercial paste from Dupont no. 7164 LuxPrint with Antimony Tin Oxide (ATO) filler. The designed structure was printed directly on a white cotton t-shirt with a weight of 180 g/m^2^.

### 2.2. Preparations

The production of homogeneous composite pastes requires thorough mixing of the functional phase in the polymer matrix. The materials were mixed with the vehicle in mortar, then stirred ultrasonically for 1 h to acquire a homogenous mixture, and rolled using three-roll-mil to disperse remaining agglomerates.

To obtain the final compositions, the luminophore and ceramic powders were dispersed in the polymer base, which was a 20 wt.% solution of TPU in the organic solvent. The conductive pastes were prepared with 15 wt.% solution of TPU. Maximum and optimal filling of the composite ensuring proper rheological properties for silver flakes was 70 wt.% and for graphene 15 wt.%. The ratio of filler to matrix in dielectric layers was 7:3.

### 2.3. Printing Process

**Figure 1:**
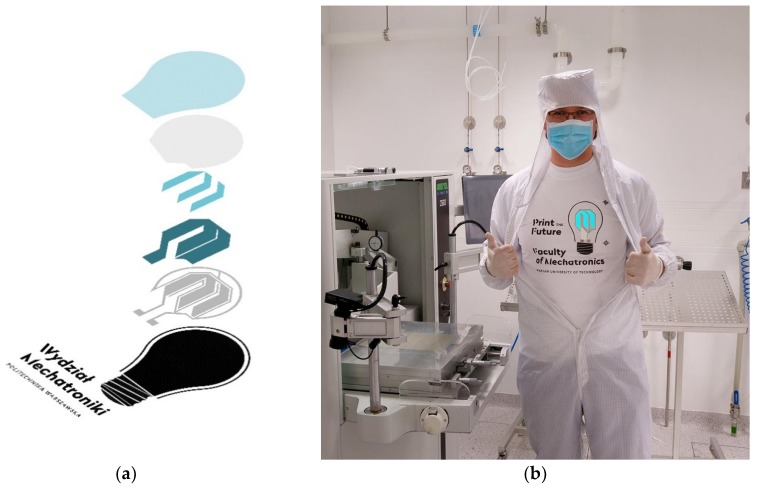
Schematic cross-section of the prepared printed electroluminescent structure (from the top: transparent protective layer, upper transparent electrode, electroluminescent layer, dielectric layer, lower electrode, protective layer), (**a**) Work results (**b**).

**Figure 2:**
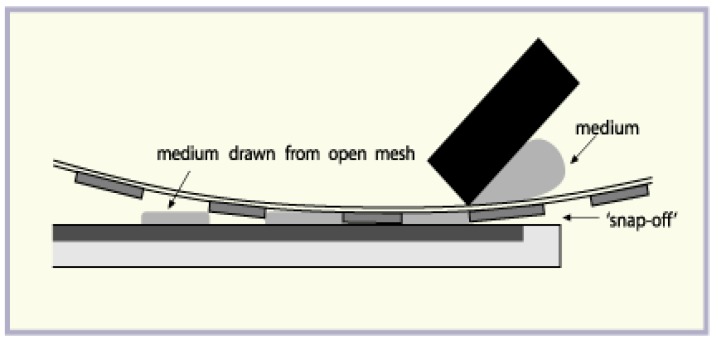
Mechanism of screen printing process.

Figure 1 shows the designed structure made during the tests. The obtained composites were screen-printed with 60 T mesh on the textile substrate in the designed order. Screen printing is the most cost-effective method for producing printing electronics. During the printing process the squeegee moves along the screen and squeezes the printing paste through the screen onto the substrate. The printing paste can only pass through an open mesh, so the deposited pattern is a projection of open mesh. The mechanism of this process is shown on Figure 2. After each printing process, the layers were dried at 150 °C for about 5 min in a tunnel infrared dryer.

After each printing process, the thickness of a single layer and of the entire structure was measured using a contact profilometer. The results are presented in Table 1.

Prepared transparent CNT electrodes with 0.5 wt.% of filler obtained optical transmissions above 70% with less than 10 kΩ/sq sheet resistivity. During the research, two types of structures were tested: A CNT-based and an ATO-based, transparent electrode, both with a silver and graphene bottom electrode.

## 3. Results

**Figure 3:**
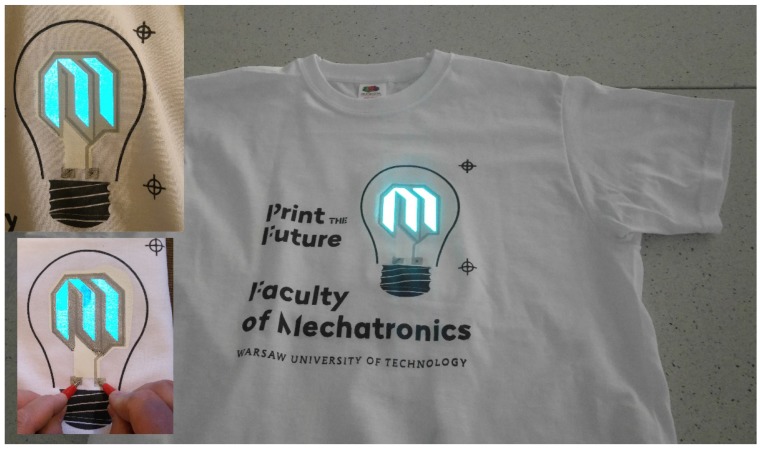
T-shirt with printed electroluminescent display. Upper left corner: Electroluminescent (EL) structure with carbon nanotube electrode, lower left corner: EL structure with an ATO-based electrode.

Functioning electroluminescent structures were successfully printed and found to emit blue light. The obtained structures are shown in Figure 3. In the upper left corner of Figure 3, the electric contacts were sewn in, using nanotube graphene wires [16], to lead the electric connection underneath the fabric. Electroluminescent structures were powered with an alternating voltage of 100 V and 1 kHz frequency. The developed transparent electrode, based on carbon nanotubes, proved its usefulness as a transparent electrode in the applications of wearable electronics.

The created multi-layer structures are protected with a transparent layer of a TPU-based carrier from the top and TPU-based layer from the bottom. Those layers isolate the whole electroluminescent (EL) structure, thereby preventing electrocution, even after applying a high voltage that causes breakdown.

Such prepared T-shirts were subjected to mechanical strength tests used for flexible structures [17,18] of 200 cycles, conducted using a fatigue machine, according to the PN-EN ISO 7854 standard (A—De Mattia method on a 10 mm diameter shaft). Those tests showed no change in EL structure. After 10 washing cycles of displays, containing a CNT-based electrode, no visible degradation of electroluminescent layer was observed. Each T-shirt was wrinkled by hand to simulate everyday wear and use. Those tests also showed no damage.

**Figure 4:**
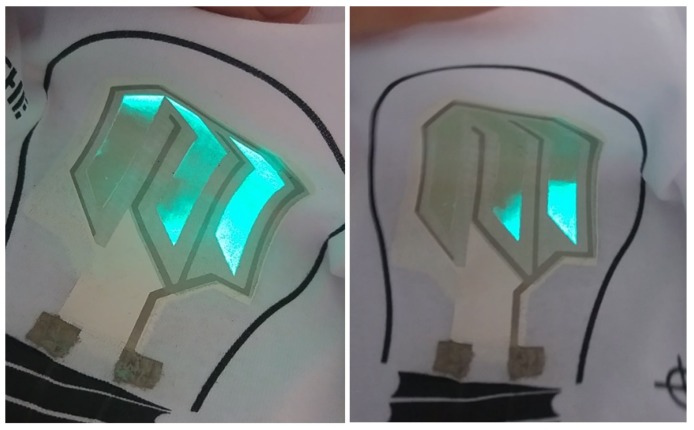
Visible degradation of two exemplary electroluminescent structures with an ATO-based electrode after first wash.

The T-shirts were subjected to washing in an automatic washing machine in the hand washing cycle at 40 °C for 30 min. Immediately after washing, the wet structures did not appear to emit light. After about 2 h of drying, the powered structures started to emit light. The displays based on a commercial electrode demonstrated some problems with uniform lighting after washing. Figure 4 shows the degradation of the ATO electrode display after the first wash.

The test for ironing the EL structure were carried out using household iron, set to 170 °C. No degradation of emitting light was noticed, and, in some cases, improvements in the EL structure was detected. This is the effect of the polymers’ recrystallization and contact improvement between layers.

**Figure 5:**
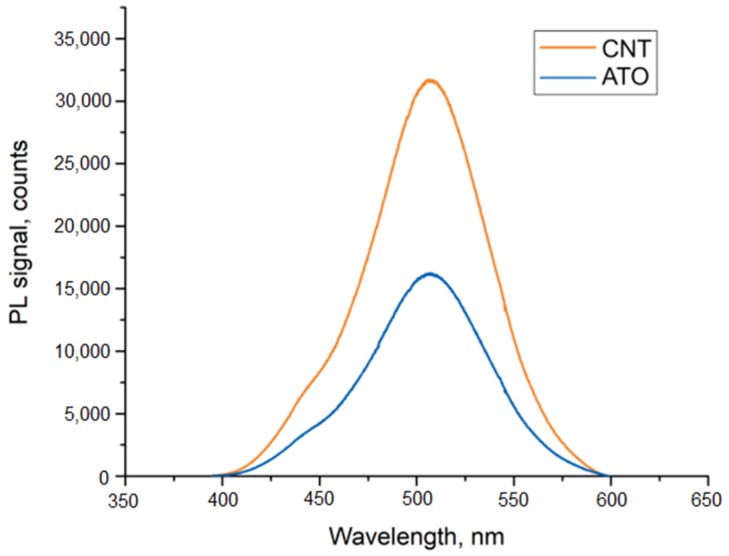
Photoluminescence (PL) Spectra for screens with different transparent electrode supplied with 234 V AC.

**Figure 6:**
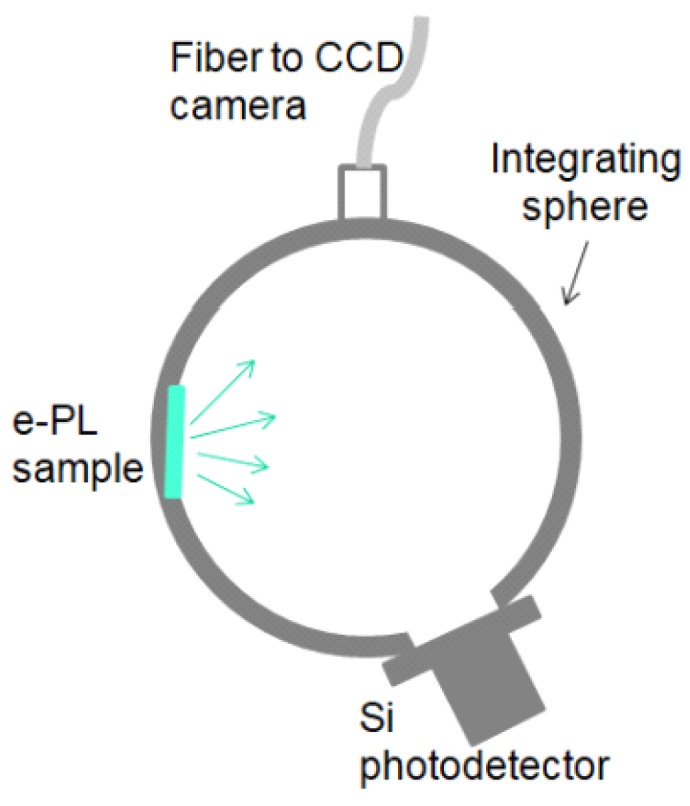
Measurement set-up for assessing the PL signal quantitatively.

**Figure 7:**
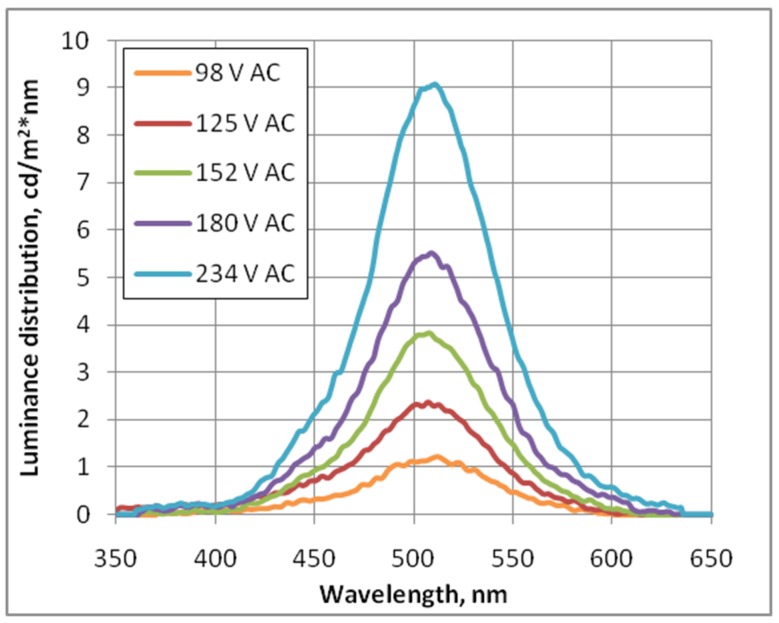
Luminance distribution for EL display with CNT based electrode supplied with different AC voltage with 1 kH frequency.

Additional tests of luminescence were performed. To analyze the measured samples, a photoluminescence (PL) signal was dispersed using a 600 lines/mm grating blazed at 500 nm and then detected by a nitrogen-cooled Si CCD. The measurement results for 2 EL displays, with different transparent electrodes, are depicted in Figure 5. To reduce the dependence of the optical set-up, the e-PL samples have been placed in an integrating sphere with an optical fiber to nitrogen-cooled Si CCD camera and a Si photodetector (Figure 6). In such set-up, the PL spectra of EL display, with CNT electrode, have been collected with different AC supply voltage of 1 kHz frequency (Figure 7).

**Figure 8:**
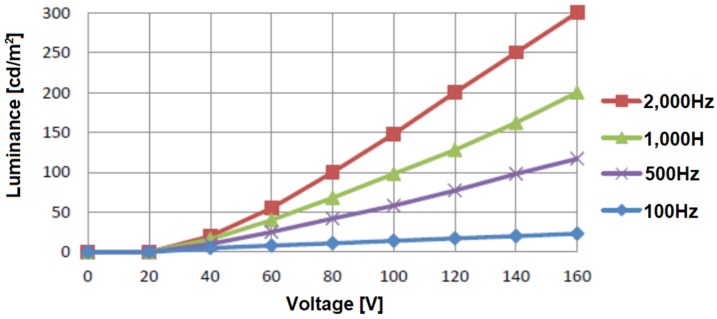
Luminance values for different AC voltages and different current frequencies.

Total luminance depends, not only on the voltage supply, but also on its frequency. The influence of voltage and current frequency on the luminance value of the display was also tested and the results are presented in Figure 8.

## 4. Discussion and Conclusions

TPU based composites using carbon nanotubes, graphene platelets, dielectric, and luminophore nanopowders were prepared. Electroluminescent structures were screen printed with those materials. The prepared composites, made with polymer base and nanomaterial particles, were confirmed to be suitable for usage in screen printing techniques. The ratio of materials in the prepared pastes was chosen, in order to match the rheological properties of the paste for the technology of screen printing.

The developed composites showed mutual compatibility. During the mechanical tests, which involved bending and crushing of the created structure, there was no noticeable delamination between the layers or the substrate. The developed polymer carrier, based on thermoplastic polyurethane, can compete with other composition carriers for stretchable applications, like poly(3,4-ethylenedioxythiophene) polystyrene sulfonate (PEDOT: PSS) or polydimethylsiloxane (PDMS). The conductive, resistive, and dielectric composites developed during the tests can be successfully used to produce several-layer structures on stretchable and textile substrates.

The upper transparent electrode based on CNT was characterized by higher transparency and 60% lower electrical resistance than the layer based on ATO. The properties resulted two-fold higher luminance of display with the CNT layer.

## Figures and Tables

**Table 1:** Results of electroluminescent display thickness measurements.

No.	Layer Name:	Layer Thickness, µm	Summary Thickness, µm
1	protective layer	12 ± 1	12 ± 1
2	lower electrode	10 ± 1	22 ± 2
3	dielectric layer (2x)	25 ± 2	47 ± 4
4	electroluminescent layer	8 ± 1	55 ± 5
5	upper transparent electrode	7 ± 1	62 ± 6
6	transparent protective layer	12 ± 1	74 ± 7
